# Impact of a package of diagnostic tools, clinical algorithm, and training and communication on outpatient acute fever case management in low- and middle-income countries: protocol for a randomized controlled trial

**DOI:** 10.1186/s13063-020-04897-9

**Published:** 2020-11-25

**Authors:** Olawale Salami, Philip Horgan, Catrin E. Moore, Abhishek Giri, Asadu Sserwanga, Ashish Pathak, Buddha Basnyat, Francois Kiemde, Frank Smithuis, Freddy Kitutu, Gajanan Phutke, Halidou Tinto, Heidi Hopkins, James Kapisi, Myo Maung Maung Swe, Neelam Taneja, Rita Baiden, Shanta Dutta, Adelaide Compaore, David Kaawa-Mafigiri, Rashida Hussein, Summita Udas Shakya, Vida Kukula, Stefano Ongarello, Anjana Tomar, Sarabjit S. Chadha, Kamini Walia, Cassandra Kelly-Cirino, Piero Olliaro

**Affiliations:** 1 Foundation for Innovative New Diagnostics (FIND) Campus Biotech, Chemin des Mines 9, 1202 Geneva, Switzerland; 2 Big Data Institute, University of Oxford, Old Road Campus, Oxford, OX3 7LF UK; 3 Oxford University Clinical Research Unit (OUCRU-Nepal), Patan Hospital, Lalitpur, Nepal; 4 Infectious Diseases Research Collaboration (IDRC), Nakasero Hill Rd, Kampala, Uganda; 5 RD Gardi Medical College, Ujjain, Madhya Pradesh 456001 India; 6 Institut de Recherche en Sciences de la Santé Clinical Research Unit of Nanoro (IRSS-URCN), Nanoro, Burkina Faso; 7 Myanmar Oxford Clinical Research Unit (MOCRU), Yangon, Myanmar; 8 Department of Pharmacy, School of Health Sciences, Makerere University, Kampala, Uganda; 9 Jan Swasthya Sahyog, Ganiyari, India; 10 London School of Hygiene and Tropical Medicine (LSHTM), Keppel Street, London, WC1E 7HT UK; 11 Post Graduate Institute of Medical Education & Research (PGIMER), Chandigarh, India; 12 Dodowa Health Research Centre, P.O. Box DD1, Dodowa, Ghana; 13 National Institute of Cholera and Enteric Diseases (NICED), Kolkata, India; 14 School of Social Sciences, Makerere University, Kampala, Uganda; 15 Dodowa Health Research Centre, P.O.Box DD1, Dodowa, Ghana; 16 FIND India, 9th Floor, Vijaya Building, 17, Barakhamba Road, New Delhi, 110001 India; 17 Indian Council of Medical Research, Division of Epidemiology and Communicable Diseases, Indian Council of Medical Research, Ansari Nagar, New Delhi, 110029 India

**Keywords:** Febrile illness, Antimicrobial resistance, Antibiotic prescription, Randomized controlled trial, Outpatient fever management

## Abstract

**Background:**

The management of acute febrile illnesses places a heavy burden on clinical services in many low- and middle-income countries (LMICs). Bacterial and viral aetiologies of acute fevers are often clinically indistinguishable and, in the absence of diagnostic tests, the ‘just-in-case’ use of antibiotics by many health workers has become common practice, which has an impact on drug-resistant infections.

Our study aims to answer the following question: in patients with undifferentiated febrile illness presenting to outpatient clinics/peripheral health centres in LMICs, can we demonstrate an improvement in clinical outcomes and reduce unnecessary antibiotic prescription over current practice by using a combination of simple, accurate diagnostic tests, clinical algorithms, and training and communication (intervention package)?

**Methods:**

We designed a randomized, controlled clinical trial to evaluate the impact of our intervention package on clinical outcomes and antibiotic prescription rates in acute febrile illnesses. Available, point-of-care, pathogen-specific and non-pathogen specific (host markers), rapid diagnostic tests (RDTs) included in the intervention package were selected based on pre-defined criteria. Nine clinical study sites in six countries (Burkina Faso, Ghana, India, Myanmar, Nepal and Uganda), which represent heterogeneous outpatient care settings, were selected. We considered the expected seasonal variations in the incidence of acute febrile illnesses across all the sites by ensuring a recruitment period of 12 months. A master protocol was developed and adapted for country-specific ethical submissions. Diagnostic algorithms and choice of RDTs acknowledged current data on aetiologies of acute febrile illnesses in each country. We included a qualitative evaluation of drivers and/or deterrents of uptake of new diagnostics and antibiotic use for acute febrile illnesses. Sample size estimations were based on historical site data of antibiotic prescription practices for malarial and non-malarial acute fevers. Overall, 9 semi-independent studies will enrol a minimum of 21,876 patients and an aggregate data meta-analysis will be conducted on completion.

**Discussion:**

This study is expected to generate vital evidence needed to inform policy decisions on the role of rapid diagnostic tests in the clinical management of acute febrile illnesses, with a view to controlling the rise of antimicrobial resistance in LMICs.

**Trial registration:**

Clinicaltrials.gov NCT04081051. Registered on 6 September 2019. Protocol version 1.4 dated 20 December 2019

## Background

The World Health Organization (WHO) considers antimicrobial resistance (AMR) to be ‘an increasingly serious threat to global public health that requires action across all government sectors and society’ [1]. It is estimated that 700,000 deaths each year are due to drug-resistant pathogens; by 2050, if no actions are taken to contain AMR, this figure is predicted to rise to 10 million deaths per year [2].

Inappropriate self-treatment and ‘just-in-case’ antibiotic prescribing practices, along with inadequate doses and treatment duration, are considered the 5main contributing causes of AMR [3, 4]. Optimizing the use of antimicrobial agents is one of the four pillars of the WHO Global Action Plan on AMR [5]. However, identifying the causative agent of an infection, or at least distinguishing between bacterial and viral infections, remains a challenge, as few diagnostics are suited to this task and are not yet affordable for widespread use in low- and middle-income countries (LMICs). Most health facilities in LMICs lack the diagnostic capacity to correctly identify the cause of an acute febrile illness, thus undermining a health worker’s ability to determine whether an antibiotic is required or not [6]. To this end, an adaptation in prescription practices needs to occur to improve patient management by healthcare staff and to change patient behaviours as well as treatment-seeking behaviours. Success will mean making significant steps toward achieving the dual goal of tackling AMR and providing universal health coverage (UHC).

Recent work in LMICs has focused on unravelling the diverse aetiologies of acute febrile illnesses especially in children [7–13]. Data from outpatient settings are scarce, with a small number of studies describing the relative distribution of bacterial, parasitic and viral agents among acutely febrile patients [7]. These diagnoses often rely on complicated serological and/or nucleic acid detection assays which are not available in primary healthcare centres, where many ill patients first present for care. Currently, there is a lack of quality-assured point-of-care tests (POCTs) for many infectious causes of fever in LMICs. Moreover, even when regulatory-approved POCTs are available, there is, in many instances, a paucity of data on their clinical usefulness within the operational contexts found in these settings [14].

WHO, in its recent technical consultation on in vitro diagnostics for AMR, has stressed the need for research and development on additional tests that can distinguish between bacterial and non-bacterial infections at primary healthcare facilities. In order to be fit for purpose, such POCTs should be rapid diagnostic tests (RDTs) such as lateral-flow tests or easy-to-use, robust diagnostic platforms that use accessible, minimally invasive clinical specimens (e.g. whole blood, urine stool or nasal swabs) [15].

Practical solutions, adapted to LMICs, are needed to provide better healthcare to patients presenting with fever, allowing healthcare workers to confidently prescribe, or not prescribe, an antibiotic based on the evidence of test results. These solutions must be applicable now, with existing tools, but also be amenable to incorporate newer and better tools, when they become available.

In recent times, attempts have been made to build antimicrobial stewardship programs into the healthcare landscape in LMICs to correct inappropriate antimicrobial prescribing and use practices [16]. This cannot happen without a proper understanding of the bacteria that are causing disease in the different countries, of how to use currently available diagnostic tests and to incorporate these tests into clinical diagnostic algorithms and how to modify practices and behaviours in order to correct ineffective management of acute febrile illnesses. The most appropriate platform for evaluating such interventions are research centres that are firmly integrated within the health system, have access to clinical care facilities and have a platform for population-based evaluation of immediate and long-term impact.

This is the basis for the intervention research study described below. All the diagnostic tests described have regulatory approval and are commercially available, but have never been field-tested together, as part of decision-making algorithms for use in peripheral health facilities in LMICs.

This study is part of the FIND AMR Diagnostics Use Accelerator, a platform which intends to stimulate studies to investigate the outcome of innovative clinical algorithms using existing diagnostic kits and to inform research into the design and implementation of effective behavioural change interventions. The project comes at an opportune time for institutions with the appropriate capacity to integrate diagnostics into healthcare delivery and to appropriately evaluate the impact.

### Study objectives

This study aims to address the following question that is based on the PICO process [17]:

In children and adolescents (+/− adults) who present to outpatient clinics or peripheral health centres in LMICs with acute febrile illness (Population), will introducing a package of available diagnostic tests, diagnostic algorithms, new clinic process flows, and training and communication for healthcare workers and patients or caregivers (Intervention), as compared with current practice (Control), improve case management of acute febrile illnesses and better target the correct use of antibiotics (Outcome)?

- Primary study objectives: to assess the impact of a package consisting of diagnostic tools, clinical algorithms, and training and communication on (1) clinical outcomes and (2) antibiotic prescriptions, compared with routine practices for children and adolescents (+/− adults in certain sites—see Table 1) presenting at outpatient clinics.
- Secondary study objectives: to assess adherence (1) to the new diagnostic algorithm by healthcare workers, (2) to prescriptions by patients/caregivers, and (3) to evaluate the safety outcomes of these practices.

**Table 1:** List of clinical trial sites, their locations and age ranges of participants

Country	Uganda	Nepal	Myanmar	Ghana	Burkina Faso	India			
Institution	Infectious Diseases Research Collaboration (IDRC)	Oxford University Clinical Research Unit-Nepal	Myanmar Oxford Clinical Research Unit (MOCRU) and Medical Action Myanmar (MAM)	Dodowa Health Research Centre	IRSS-DRCO/Clinical Research Unit of Nanoro (CRUN)	Chhattisgarh: Jan Swasthya Sahyog (JSS)	Chandigarh: Post Graduate Institute of Medical Education & Research (PGIMER)	Madhya Pradesh: RD Gardi medical college	West Bengal: National Institute for Cholera and Enteric Diseases (NICED)
Number of recruiting sites	3	1	4	2	2	4	1	1	2
Nature of recruiting sites	Health centres in three regions (two rural and one peri-urban) with varied geography and malaria endemicity, outside Kampala	Large, urban teaching hospital in Kathmandu	NGO supported, stand-alone clinics located in peri-urban areas, and one hospital in Yangon	one regional referral hospital in greater Accra, and one rural hospital	Rural, health centres 90 km from the capital	Rural health centres	Teaching hospital for urban and rural patients	Urban medical college	Small clinics located in urban slums in Kolkata
Age ranges included	all ages > 1 year	all ages > 6 months	all ages > 1 year	6 months to 18 years	6 months to 18 years	6 months to 18 years	all ages > 6 months	all ages > 3 months	6 months to 18 years

### Explanation for choice of comparators

For this programmatic study, routine clinic processes will serve as the comparator to the intervention package. However, different to routine practice, both arms will have a day 7 follow-up visit to the health facility.

## Design and methods

### Study settings

The study sites were identified through two calls for partners: one for Africa and Asia (excluding India) [18], and one for India only [19]. Applications were reviewed by two respective selection committees who recommended three sites in Africa, two in Asia and four in India; after appropriate due diligence the sites were chosen. Information on the sites is summarized in Table 1.

### Study design

The study design captures a wide range of qualitative contextual outcomes and quantitative empirical outcomes in order to address the various aspects of the core research question.

Overall, this is a prospective, comparative, multicountry and multisite, open-label, two-armed, 1:1, randomized-controlled trial which will compare the impact of the intervention package (POCTs, clinical algorithm, clinic process flow, and training and communication) on clinical outcomes and antibiotic prescription patterns in febrile patients with the current practice. All sites have adopted a common protocol that reflects differences in patterns of infection and behaviours at each site and in each geographical area.

As this is principally a site-level study, sample sizes have been generated to provide the required statistical power for each individual site. Analyses will be conducted at two levels: first at the site level on individually randomized study participants, and secondly, on the combined studies as an aggregated-data meta-analysis across all sites, with the site as the unit of analysis.

### Study participants

The study will recruit children and adolescents (and adults at some sites—Table 1) attending study site outpatient facilities who present with an acute febrile illness (current or within the past 7 days) defined as fever with no focus or suspected respiratory tract infection (RTI).

### Eligibility criteria

The study will include patients who meet all of the following criteria:

- Aged within the eligible range for the site (Table 1);
- Presenting with acute fever defined as having a temperature of > 37.5 °C or a history of fever within the last 7 days with no focus, or with suspected RTI;
- Lacking symptoms and signs of severe illness that require hospital admission or referral as assessed by the study clinicians;
- Informed consent for children under 18 years of age provided by parent/guardian; patient assent required for adolescents 18 years or older;
- Willing to provide blood and other samples and to adhere to study procedures explained in the information sheet/consent forms following the protocol;
- Available and willing to return for follow-up visit at the health facility on day 7 (+/− 2 days)

Patients are excluded from the study if they fail to meet any of the inclusion criteria listed above.

Data will also be gathered from healthcare workers and patients/caregivers for the qualitative components, where consent is provided.

### Qualitative pre-intervention study

The qualitative study will be conducted to understand the contextual factors and behavioural determinants affecting adherence to prescriptions by patient/caregivers and the associated communication about prescriptions by healthcare workers. The findings will inform the Training and Communication package which will be implemented as part of the clinical intervention arm consisting of diagnostic algorithms, POCTs, prescribing decision trees, clinic process flow, training and communication on adherence to prescription. Further research using the capacity, opportunity and motivation (COM-B) model [20] together with the Theoretical Domains Framework (TDF) [21] during the recruitment phase will investigate the wider behaviour change implications of adherence to prescription beyond the Training and Communication package, as well as the long-term effects of behavioural determinants for healthcare workers who choose to use the clinical algorithms, new point-of-care RDTs and associated prescribing practices.

### Interventions

**Fig. 1:**
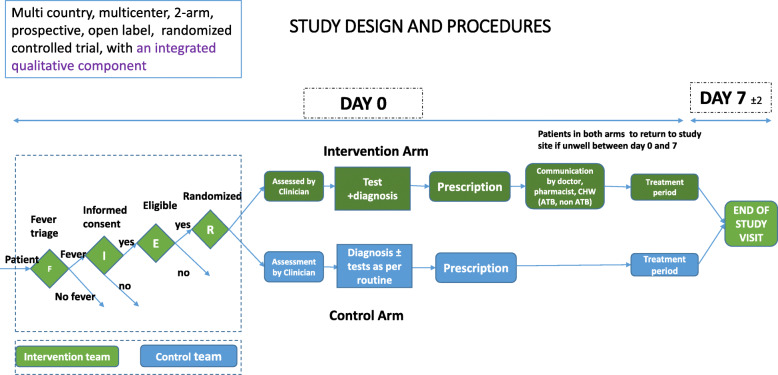
Outline of diagnostic and clinical algorithm. RSV, respiratory syncytial virus; GAS, group A streptococci; CRP, C-reactive protein; WBC/DIFF, white blood cells total counts/differential counts; Rx, treatment; ATB, antibiotic; Y, prescribe antibiotic; N, antibiotic prescription not warranted; P, antibiotic prescription possible if clinically indicated

The trial intervention will consist of (1) diagnostic tests (Table 2), (2) diagnostic and clinical algorithm (Fig. 1), (3) training and communication of healthcare workers and patients/caregivers and (4) clinic process flow.

**Table 2:** Pathogen-specific point-of-care (POC) tests

Pathogen	Type of test
Dengue virus	Lateral flow RDT: detects Dengue virus NS1 antigen and IgM (and IgG) from serum or whole blood
*Streptococcus pyogenes*	Lateral flow RDT: detects group A streptococcal antigen from throat swabs
*Salmonella enterica* serovar Typhi	Lateral flow RDT: detects *Salmonella typhi*-specific IgM from serum or whole blood
*Orientia tsutsugamushi* (scrub typhus)	Lateral flow RDT: detects *Orientia tsutsugamushi* antibodies (IgG, IgM, and IgA) in human serum, plasma, whole blood
Influenza virus	Lateral flow RDT: detects influenza virus type A, type B and A(H1N1) pandemic antigens directly from nasal/throat/nasopharyngeal swab or nasal/nasopharyngeal aspirate
Chikungunya virus	Lateral flow RDT: detects Chikungunya virus IgG/IgM antibodies in serum, plasma or whole blood
*Streptococcus pneumoniae*	Lateral flow RDT: detects *S. pneumoniae* antigen in the urine of patients with pneumococcal pneumonia
Respiratory syncytial virus	Lateral flow RDT: detects respiratory syncytial virus (RSV) fusion protein antigen in nasal wash and nasopharyngeal (NP) swab specimens
*Leptospira interrogans* (leptospirosis)	Lateral flow RDT: detects *Leptospira interrogans* IgM antibodies from urine
*Plasmodium Sp*. (malaria)	Lateral flow RDT as per national guidelines

#### Diagnostic tests

The diagnostic tests to be used in the intervention package (Table 2) will be selected based on local needs.

Non pathogen-specific POCTs:

- White blood cell total and differential counts (WBC/diff)
- Urine dipstick
- C-reactive protein (CRP)

#### Diagnostic and clinical algorithm

Healthcare workers will follow a country-specific algorithm for cases in the intervention arm, which takes into account the specific tests to be used at the site. A typical clinical algorithm is outlined in Fig. 1. Notably, the decision on pathogen-specific diagnostic tests to use in each case was based on clinical presentation, which we have grouped into respiratory and non-respiratory signs and symptoms. In addition, clinical decisions in the intervention arm will be supported by CRP and WBC/diff POCT.

#### Training and Communication package

Communication messages about adherence to prescription are delivered by healthcare worker(s) to the study patient or caregiver in the intervention arm. The method of delivery of the communication message will be site-specific, depending on the roles of different types of healthcare workers, and behavioural determinants identified in the qualitative analysis. Training is provided to healthcare workers to deliver the communication messages; together, the training and delivery of the messages make up the Training and Communication package developed for each site based on findings from the qualitative work identifying behavioural drivers of adherence to prescriptions. The delivery of the core communication message will be personalized to the individual patient (e.g. adjusting emphasis, but not message content), based information collected from the patient before clinical assessment.

#### Clinic process flow

At the majority of sites, the clinic process flow described below, closely follows the normal clinic flow, only with minor changes to speed up the process, such as mechanisms to minimize queuing.

### Participant flow

**Fig. 2:**
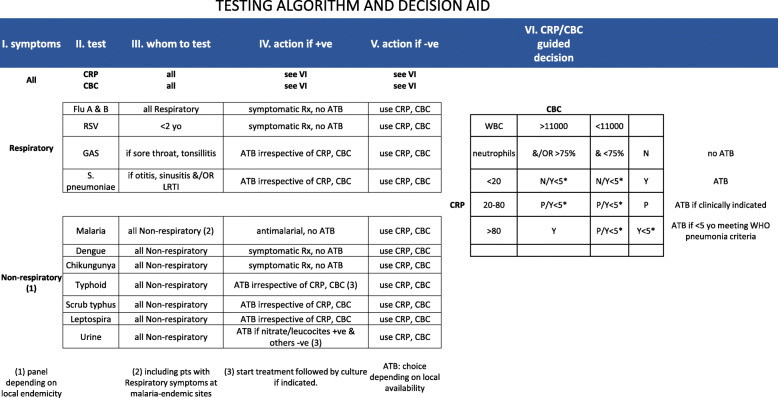
Study participant flow

The participant flow is shown in Fig. 2. Patients presenting at the clinic will be pre-screened for fever and those meeting the study eligibility criteria will be enrolled in either the control or intervention arm of the study by simple, 1:1 randomization. Participants in both arms will be seen by clinicians who will collect patient histories and conduct clinical examinations.

Patients in the control arm will follow routine diagnostic and treatment procedures for acute febrile illness as outlined in each clinic. Patients in the intervention arm will first be asked to provide information on their attitude towards the use of antibiotics, and then undergo a provisional diagnosis (respiratory/non-respiratory). Samples will be taken from patients to be performed using POCTs and any other tests based on the provisional diagnosis and algorithm. An additional communication package to influence adherence to prescription will be provided by the study team (clinicians, pharmacist and/or nurses) before the patient leaves the facility.

All patients in both the intervention and control arms will be followed up on day 7 (+/− 2) in the health facilities, to reassess their health status and prescription adherence.

Patients who do not improve following the day of enrolment will need to visit the health facilities making an unscheduled visit before the day 7 (+/− 2) follow-up visit for further assessment and management.

The package of POCTs provided in the intervention arm is considered to be an addition to tests provided in routine care. Therefore, where site routine protocols include the use of blood culture or other laboratory tests, these will also be conducted in the intervention arm (along with the normal practice in the control arm). Patients will return to the clinic for review of their prescription in light of these test results, following the normal schedule.

### Biological specimens

Biological samples will be handled as outlined below:

- Two millilitres of venous blood samples will be taken from adult patients and children over 5 years of age, as per the Seattle Children’s Hospital Guidelines (no venous sample will be taken from patients weighing less than 4 kg and less than 5 years for safety reasons).
- For children under 5 years of age, finger prick blood will be taken instead of 2 mL venous blood. The blood sample will be used for PoC tests (CRP, Hb and white cell differential and pathogen specific rapid tests) as per clinical algorithms and SOPs adapted from product manufacturers’ user manuals.
- A nasopharyngeal swab will be taken from patients who need to be tested for influenza or respiratory syncytial virus or both as per clinical algorithm and SOPs adapted from product manufacturers’ user manuals.
- A throat swab will be taken from patients with pharyngitis/pharyngitis and tested as per clinical algorithm and SOPs adapted from product manufacturers’ user manuals
- A urine sample will be taken for patients with suspect of urinary tract infection or who need *Streptococcus pneumoniae* urine antigen tests (adults) and tests performed as per SOPs adapted from product manufacturers’ user manuals.
- Repeat or unscheduled samples may be taken for safety reasons or for technical issues with the samples.
- All unused samples will be destroyed according to national or site-specific sample destruction guidelines.
- No biological samples will be stored.

### Strategies to improve and assess adherence

Patients and caregivers in the intervention arm (but not the control arm) will receive a communication message in support of adherence to the prescription, based on the local research findings described above. The method of delivery of the communication message will be site-specific, depending on the roles of different types of healthcare workers, and the behavioural determinants identified in the qualitative analysis.

The delivery of the core communication message will be personalized to the individual patient (e.g. adjusting emphasis, but not message content), based on a small amount of information collected from the patient before clinical assessment.

All patients in both the intervention and control arms will be followed up on day 7 (+/− 2) in the health facilities to reassess their health status and adherence to prescription. Information on adherence to prescription will be collected using qualitative in-depth interviews with the patient and through pill counts (participants/caregivers will be asked to bring back packaging and remaining medicine on day 7). This includes assessing the behaviour of patients who were not prescribed medication to find out if they obtained any antibiotics or any other treatment, the reasons for this and where the antibiotics or other treatment were obtained.

All enrolled participants (and caregivers, in case of children) will be reminded prior to departure on day 0 to visit the health facilities if their illness worsens before day 7. All participants who do not return on day 7 will be followed up via telephone to assess their well-being and the need to return to the clinic for the end of study assessment. Where feasible and where consent was provided by study participants at enrolment, the investigators will conduct home visits if the patient is unable to return to the clinic for the day 7 follow-up visit.

Adherence to the diagnostic algorithm will be evaluated by reviewing the case report forms (CRFs) to assess algorithm-driven use of POCTs and antibiotic prescriptions.

### Study outcomes

The primary outcome measures are the point estimates of:

1. The proportion of cases of acute febrile illness with favourable outcome (defined as being alive + no fever + resolution of day 0 symptoms) on day 7, in both arms.
2. The proportion of antibiotic prescriptions for acute febrile illness in the two arms. Specifically, the study intends to detect a reduction (at least 30% from the baseline) in unnecessary antibiotic prescribing, without adversely affecting clinical outcomes. While antibiotic prescription rates are known at the study sites, outcomes are not currently known because patients are not routinely followed-up.

The secondary outcomes are:

1. Proportion of patients prescribed antibiotics at clinics who describe adherence to prescription on day 7.
2. Proportion of healthcare workers who adhered to new algorithm.
3. Proportion of participants experiencing adverse events (see description in Safety reporting section).

### Participant timeline

The schedule of events is described in Table 3.

**Table 3:** Timeline for study participants

Schedule of events			
	Day 0 visit	Day 7 visit (+/− 2 days)	Unscheduled visit
Informed consent and enrolment	x		
Demographic data collection	x		
History/physical examination	x	x	x
Diagnostic tests	x		
Treatment prescription	x		x
Patient adherence evaluation		x	x
Collection of qualitative data	x	x	
Delivery of communication messages	x		
Adverse event monitoring (see Safety reporting section)		x	x

### Sample size estimation

The sample size calculations were based on criteria that would allow evaluation of antibiotic prescribing at each site.

**Fig. 3:**
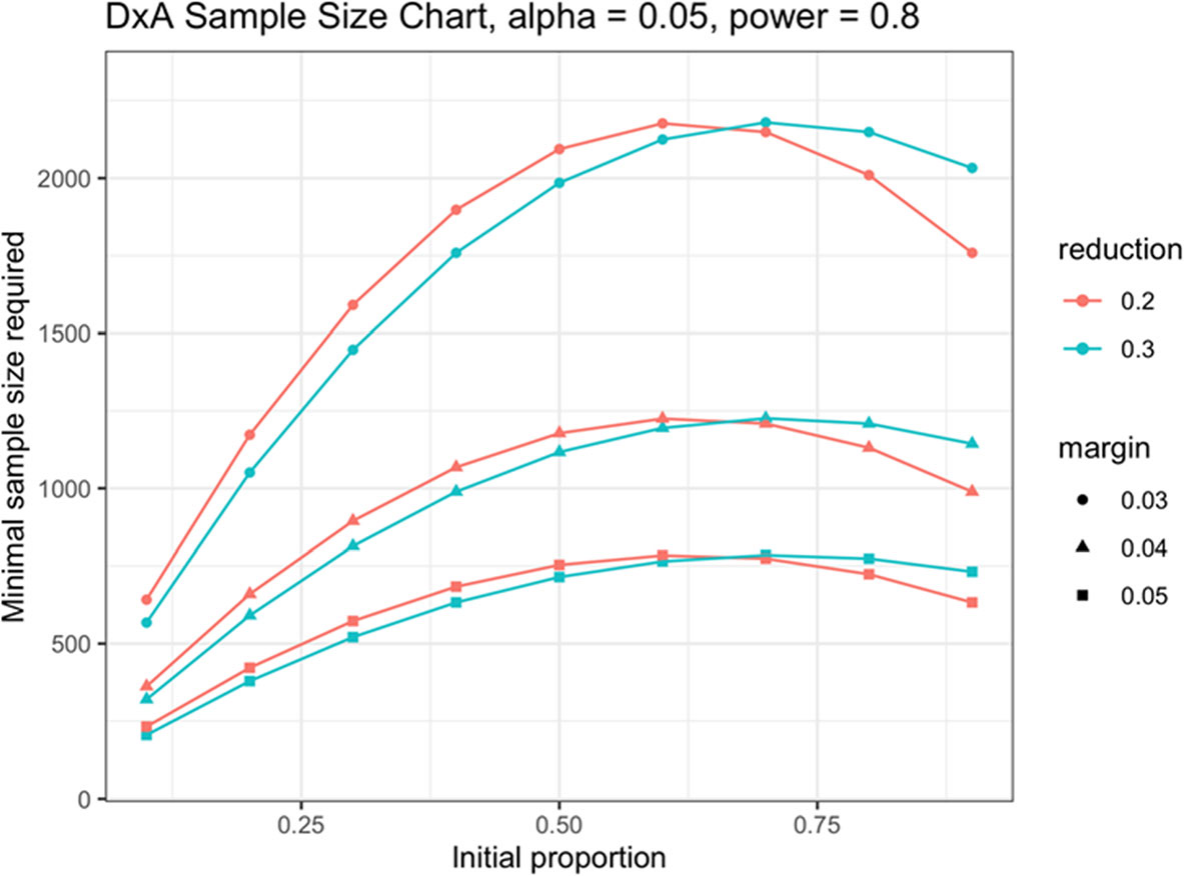
Sample size chart

The sample size was calculated for each country in order to detect a 30% difference (with a 95% confidence interval of +/− 5%) in the antibiotic prescription proportions compared with the initial proportion without the intervention, with a power of at least 80% to detect such a precision estimate (Fig. 3). The minimal theoretical sample size obtained for each site [20] has been increased by 10% to allow for loss to follow-up.

Estimates of the current proportion of patients prescribed antibiotics for acute febrile illnesses at different clinics in each country study were used as the basis to calculate the required sample size (Table 4).

**Table 4:** Baseline antibiotic prescription rates for acute febrile illnesses

Country	Expected antibiotic prescription rate in control arm (based on historic prescribing)	Reduction in prescribing	*N* per arm for children/adolescents	*N* per arm for adults	Total sample size (both arms, including losses to follow-up)
Nepal	55%	30%	1760	1760	3520
Uganda	73%	30%	1200 (combined)		2400
Ghana	43%	30%	1383	n/a	2766
Burkina Faso	77%	30%	859	n/a	1718
Myanmar	43%	30%	440	440	1760
India JSS	50%	30%	864	n/a	1728
India PGIMER	50%	30%	864	864	3456
India RD Gardi	20% (children), 40% (adults)	30%	553	831	2768
India NICED	50%	30%	880	n/a	1760
Total					21,876

Sample sizes are considered a minimum. Enrolment will continue after the sample size has been reached, through the 12 months of the intervention, to allow for seasonal variations.

### Strategies for achieving adequate participation enrolment

We estimate, based upon clinic registries for the previous 1 or 2 years, that there will be adequate enrolment of participants in all of the sites.

### Assignment of interventions

Enrolled patients will be randomized into either the intervention or the control arm. Patients in the intervention arm will be managed with a package of interventions (as detailed above) and patients in the control arm will be managed as per routine clinical practice.

Patients will be randomized centrally, by site, into the intervention or control arm in a 1:1 allocation ratio. Randomization codes will be prepared by the trial data manager based on the sample size estimates for each site and will be sent prior to commencement of enrolment to each site.

### Blinding and masking

Not applicable as this is an open label study.

### Data management

All anonymized clinical participant data relating to the trial will be recorded in electronic CRFs created on the study sponsor’s online clinical trials platform (OpenClinica Enterprise Edition *version 4.0*). The sponsor will provide training on CRF completion prior to the start of the study. Electronic data capture (EDC) will be performed via eCRFs on android tablets in offline mode, to facilitate data entry at peripheral sites with limited internet connections, and synchronized daily at site research offices with verified, reliable internet connections. Paper CRFs will be available as back-up. Only authorized study personnel will have access to the CRFs. Consent forms containing patient identifying data will be retained at each site and kept in secure lockable cabinets in a secure room with restricted access.

Records and documents, including signed informed consent forms (ICF) pertaining to the conduct of this trial will be retained by the principal investigator for 10 years after trial completion, unless local regulations or institutional policies require a longer retention period.

### Data quality

Training on the protocol, Good Clinical Practice (GCP), Good Clinical Laboratory Practice (GCLP) and the use of the diagnostic tests will be provided by the sponsor and University of Oxford. A laboratory manual, which describes all of the sample testing procedures, will be provided by FIND prior to the commencement of the trial. Detailed training on the EDC system will be provided by the data manager prior to first participant enrolment at each site. All of the POCT results will be photographed following a standard operating procedure. These photographs will be uploaded into the CRF to ensure the quality of the POCT data being recorded in the database and so that corrective procedures can be followed should there be any discrepancies between the laboratory staff and the monitor, since a patient’s ongoing care is based on these results. The sponsor will perform risk-based monitoring of this trial, and associated quality control checks in line with ICH GCP, and all applicable regulatory requirements.

Training on qualitative methods of data collection, analysis and use of the behaviour change wheel [21] process will be provided by the University of Oxford and/or onsite social science leads.

### Statistical methods

We have a dedicated SAP that is currently under review that will cover the statistical analysis in great detail, focusing not only on the country-specific analyses, but also on the overall analysis from the combination of the data from all countries. We foresee the analysis to need some country-specific adjustments. However, here is an overview of the analyses we will perform.

### Populations for statistical analyses

Table 5 describes the study populations for the purposes of analyses.

**Table 5:** Populations for statistical analyses

Population	Description
1. Enrolled	All participants who sign the ICF
2. Randomly assigned to trial intervention (RAI)	Randomized to either intervention or control arm
3. Modified intent to treat (mITT)	Participants in RAI with partial data (but not fully compliant with the protocol)
4. Evaluable per protocol population (PP)	All participants who fully complied with the protocol
5. Safety population for analysis	All participants randomly assigned to trial intervention. Participants will be analysed according to the intervention they actually received.

### Descriptive statistics

Descriptive statistics tables will be generated to summarize the characteristics of the participants. The number of participants included and excluded will be reported. Among the included participants, the information will be broken down by site, sex, and age group. Results will be reported either in absolute numbers (e.g. number of subjects in a group) or summarized by mean, median, standard deviation, minimum, maximum and quartiles.

### Efficacy analyses

The point estimates of the study outcomes (percentage of antibiotic use, percentage reduction from baseline), with a 95% confidence interval based on the Wilson score interval, will be calculated for (1) clinical outcome (favourable or unfavourable), (1a) between-arms and (1b) between compliant and compliant patients within arms and for (2) antibiotic use, also (2a) between-arms and (2b) within-arms.

### Analysis of endpoints

The endpoints of the trial will be evaluated as follows:

- The proportion of cases with a favourable outcome in the two arms, in the mITT and PP population
- The proportion of compliant cases with a favourable outcome in the PP population
- The proportion of inappropriate antibiotic prescription in the PP population
- The antibiotic prescription rate in the two arms, in the mITT and PP populations
- The proportion of antibiotic/no antibiotic prescriptions with favourable/unfavourable outcomes

### Other analyses

We will calculate the combined proportion of compliant participants with favourable outcomes and non-compliant participants with unfavourable outcomes within each arm.

Additionally, the ratio of the proportion of compliant participants with a favourable outcome versus the proportion of non-compliant participants with an unfavourable outcome will be reported as relative risk.

There is no planned interim analysis.

### Handling of missing data

Missing and invalid data for key measures that contribute to the evaluation of each of the outcomes will be reported in summary tables and will not be imputed. The primary outcomes do not involve quantitative assessments, and therefore, we feel it would not be appropriate to input data. Data from participants for whom partial data are available will be included in the analysis of specific outcomes, provided the data allow to evaluate them (e.g. adherence to prescription).

### Data and safety monitoring board

Given that the clinical decision to prescribe or not prescribe antibiotics in the intervention arm of the study will be guided by the intervention package, there is a possibility that the diagnostic algorithm may fail to detect a need for antibiotics in some participants, thereby exposing them to the risk of getting worse from disease. It is also possible that routine procedures might expose patients to risks, such as those related to inappropriate use of antibiotics or missed diagnosis, which we will monitor, but we will not change the routine care for the patients in the control arm during the trial.

Therefore, we have established a data safety monitoring board (DSMB), composed of experts, many of whom are based in LMICs with experience in paediatrics, tropical infectious diseases, statistics, clinical trials and social science work.

The DSMB will evaluate ongoing safety data, assess the risks and benefits and give recommendations on whether to continue, amend or stop the trial. The DSMB will review, at agreed intervals, the following data as they relate to the study progress and participant safety:

- Number of screened patients, by study site
- Number of included patients, by study site and by study arm
- Patients’ baseline characteristics
- Number of patients withdrawn and lost to follow-up, by study site and by study arm and reasons for withdrawal
- Number of serious adverse events (deaths and hospitalisations) by study arm

### Safety reporting

ICH definitions of adverse events (AEs) in this study will be interpreted in light of the lack of testing for any new diagnostic or medicinal product.

We will capture untoward events occurring during the period under observation in order to understand if either approach (current practice vs. new package of interventions) might be associated with a risk to the patient’s welfare (e.g. unnecessary use of antibiotics that could cause AEs; conversely, a practice that denies a patient a treatment that was otherwise warranted).

Non-serious AEs are to be reported on the CRF. In addition, each site principal investigator will be responsible for reporting serious adverse events (SAE) to the sponsor (FIND) within 24 h as well as to the Ethics Review Committee with which they are associated, depending on the local requirements.

### Ethical approvals

The overall protocol has been approved by the Oxford University Tropical Research Ethics Committee (OxTREC number 52-19). Each of the country-specific protocols is also approved by national and/or institutional ethics committees in Burkina Faso, Ghana, Uganda, India, Nepal and Myanmar* (*final document pending at the time of writing).

### Informed consent

Informed consent will be obtained by the healthcare workers/social scientists from all participants and/or their legally authorized representatives/caregivers prior to their participation in the study. Patient information sheets and consent forms have been developed in English and translated into local languages within the respective study sites. Participants or their parents/caregivers will be required to sign and date a statement of informed consent that meets the requirements of the ethics review committees.

A copy of the ICF(s) will be given to the participant or the participant’s parents/caregivers.

### Protocol amendments

We will share information on important protocol modifications (e.g. changes to eligibility criteria, outcomes, analyses) with the investigators, REC/IRBs, trial participants, trial registries, journals and regulatory agencies.

### Confidentiality

Participants will be assigned a unique identifier or study number. Any participant records or datasets that are transferred to FIND will contain the identifier only; participant names and any information which would identify the participant will not be transferred.

The patient study number will be on all labels, data collection sheets and in the database to maintain confidentiality, including in the CRF. All documents relating to the clinical study will be stored securely electronically within OpenClinica and will only accessible by study staff and authorized personnel. The study will comply with the General Data Protection Regulation (GDPR), which requires that personal data must not be kept as identifiable data for longer than necessary for the purposes concerned. All qualitative data will be stored at the sites.

### Access to data

Only trial staff in country will have access to the data for each country. Only FIND as study sponsor, and Oxford, as study collaborator, will have access to the anonymized data collected at all of the sites. FIND will perform the meta-analysis following the statistical analysis plan.

### Ancillary and post-trial care

The study aims to evaluate clinical outcomes and antibiotic prescription rates for acute fevers 7 days post-enrolment and does not provide specific therapeutic interventions beyond the marketed and approved medications which will be prescribed by the treating physicians based on either routine practice or the new diagnostic package. For participants who experience complications from an acute febrile illness in either arm of the study, resources will be provided to manage these, in line with the national guidelines in each country.

### Dissemination policy

The publication policy follows the ICMJE guidelines [22]. Both the protocol and the study outcomes will be published in peer-reviewed, open-access journals, and the data will be made available. We intend to publish both the individual study results and the aggregated-data meta-analysis. We will also publish the qualitative methodologies and results for the study as a whole, as well as for the individual studies.

We also intend to prepare policy briefs and summaries as required for policy makers at both country and international level, in order to inform guideline development and policy.

## Discussion

The FIND AMR Diagnostics Use Accelerator is a platform to evaluate a package of interventions and provide evidence to inform policy change that can positively impact antimicrobial resistance (AMR) and contribute to universal health coverage (UHC).

This article presents the rationale, methods, and procedures of a large multicounty clinical trial in which we aim to investigate whether adopting a package of available POCTs, clinical diagnostic algorithms, clinic process flow adjustments, and training and communication activities can result in improved care of acute febrile illness and more rational antibiotic prescribing and use.

Many published studies that evaluate infectious febrile illnesses are conducted over only a few months, and therefore do not adequately account for seasonal variations in such illnesses [6], representing a major methodological weakness. We aim to address this by ensuring a 12-month study period which captures seasonal variations in infectious diseases in the study countries.

The study will focus on cases presenting at peripheral health facilities and hospital outpatient departments as this is generally the first encounter that patients have with the healthcare system where antibiotics can be prescribed. These cases represent a sample of community-acquired infections which are serious enough to seek treatment, but not serious enough to require hospitalization. The study in some sites targets children and adolescents as a recognized vulnerable group, while other sites have included adult participants for broader population representation (see Table 1).

There are a limited number of POCTs that could be deployed and used at peripheral health centres to help differentiate between bacterial and viral infections (host biomarkers) or diagnose a specific bacterial or viral agent (pathogen-specific tests). To identify suitable candidates, we undertook a landscape scoping analysis of available POCTs and matched this analysis with the prevalent infections reported by the participating study sites.

It is anticipated that conditions will vary in different study settings, and the overall package of interventions has been adapted to local background infection profiles and practices. Therefore, the study has a common template protocol, which is tailored to the conditions of participating study sites. This will allow to draw both locally-relevant and generally-applicable conclusions as to the utility of the proposed package of interventions.

The FIND AMR Diagnostics Use Accelerator could potentially serve as a pathfinder for generating evidence that will inform policy change and lay the groundwork for a robust route to diagnostic uptake and thus positively impact patient care globally.

### Trial status

The study protocol reflects approved version 1.4 from 20 December 2019. Recruitment at the first site will commence in July 2020 and is expected to be completed across all sites in December 2021.

## Data Availability

The datasets used and/or analysed during the current study are available from the corresponding author upon reasonable request.
